# Development of Monolithically Grown Coaxial GaInN/GaN Multiple Quantum Shell Nanowires by MOCVD

**DOI:** 10.3390/nano10071354

**Published:** 2020-07-10

**Authors:** Kazuma Ito, Weifang Lu, Naoki Sone, Yoshiya Miyamoto, Renji Okuda, Motoaki Iwaya, Tetsuya Tekeuchi, Satoshi Kamiyama, Isamu Akasaki

**Affiliations:** 1 Department of Materials Science and Engineering, Meijo University, Nagoya 468-8502, Japan; 203428004@ccalumni.meijo-u.ac.jp (K.I.); n-sone@koito.co.jp (N.S.); 203428030@ccalumni.meijo-u.ac.jp (Y.M.); 203428012@ccalumni.meijo-u.ac.jp (R.O.); iwaya@meijo-u.ac.jp (M.I.); take@meijo-u.ac.jp (T.T.); skami@meijo-u.ac.jp (S.K.); akasaki@meijo-u.ac.jp (I.A.); 2 Koito Manufacturing Co., LTD., Tokyo 108-8711, Japan; 3 Akasaki Research Center, Nagoya University, Nagoya 460-8601, Japan

**Keywords:** monolithic growth, multi-color emission, coaxial MQS nanowires, In incorporation

## Abstract

Broadened emission was demonstrated in coaxial GaInN/GaN multiple quantum shell (MQS) nanowires that were monolithically grown by metalorganic chemical vapor deposition. The non-polar GaInN/GaN structures were coaxially grown on n-core nanowires with combinations of three different diameters and pitches. To broaden the emission band in these three nanowire patterns, we varied the triethylgallium (TEG) flow rate and the growth temperature of the quantum barriers and wells, and investigated their effects on the In incorporation rate during MQS growth. At higher TEG flow rates, the growth rate of MQS and the In incorporation rate were promoted, resulting in slightly higher cathodoluminescence (CL) intensity. An enhancement up to 2–3 times of CL intensity was observed by escalating the growth temperature of the quantum barriers to 800 °C. Furthermore, decreasing the growth temperature of the quantum wells redshifted the peak wavelength without reducing the MQS quality. Under the modified growth sequence, monolithically grown nanowires with a broaden emission was achieved. Moreover, it verified that reducing the filling factor (pitch) can further promote the In incorporation probability on the nanowires. Compared with the conventional film-based quantum well LEDs, the demonstrated monolithic coaxial GaInN/GaN nanowires are promising candidates for phosphor-free white and micro light-emitting diodes (LEDs).

## 1. Introduction

White light-emitting diodes (LEDs), which cover the full spectral range of visible light, are regarded as an inevitable technology in illumination, micro-display and visible light communication systems [1,2,3]. GaInN with a broad bandgap tunability from 0.69 eV (InN) to 3.4 eV (GaN) is one of the most popular materials for white LEDs [4,5]. Commercialized white light LEDs combine efficient blue LEDs [4,6,7,8] with a yellowish phosphor layer such as cerium-doped yttrium aluminum garnet (YAG: Ce) [9,10,11,12,13,14,15]. However, phosphors doped with rare-earth elements are not only expensive, but also increase the Stokes energy conversion loss. Furthermore, the phosphors are degraded by heat accumulation, the response rate is slow, and the correlated color temperature (CCT) has low stability [9,16]. Accordingly, a monolithic white LED that emits across the visible light range without phosphor converters is highly demanded. Nevertheless, long-wavelength emission is difficult to achieve from GaInN/GaN multiple quantum well structures, owing to the degradation of crystalline quality and the large strain-induced piezoelectric field [17]. Recent studies have achieved different emission wavelengths from three-dimensional structures fabricated by local strain engineering [18,19,20]. Using the top-down etching method, the authors of reference [21] fabricated monolithic LEDs combining microstructure and nanostructures of GaInN/GaN, which emit distinctive blue-green-yellow light with a color rendering index (CRI) of 41. However, the fabrication process (even when performed by surface passivation or thermal annealing) can also severely damage the surface and significantly decrease the emission efficiency [22].

The bottom-up growth of GaInN/GaN core–shell structures has received increasing attention in recent years, as it promises to reduce the compressive strain and internal electric field in LEDs [23,24]. Selective-area growth improves the quality of GaInN/GaN nanostructures, and the control of the indium (In) composition on different facets enables multi-colored emission. These properties are important for realizing monolithic white LEDs on a single chip. Many attempts to fabricate phosphor-free white light LEDs using selectively grown GaInN/GaN nanostructures have been reported [25,26,27]. Coaxial GaInN/GaN dodecagonal ring structures have achieved white LEDs with a low CCT (4500 K) and high CRI (81), but their effectiveness depends on the thicknesses of GaInN/GaN layer and the In contents at multiple facets [28]. Yellow-white emission was reported from composition-graded GaInN/GaN nanocolumns that were sequentially grown at 700 °C, 675 °C and 650 °C via plasma-assisted molecular beam epitaxy [27]. Recently, white LEDs based on monolithically integrated nanowires (NWs) were formed by combining multiple n^++^-GaN/Al metal/p^++^-GaN tunnel junction structures with coaxial GaInN/GaN NWs structures [29]. However, the emission wavelength flexibility (CRI) in the resulting structures was poor in these methods. Alternatively, the emission wavelength in NW structures with same pitch can be tuned by varying the NW diameter, because more adatoms diffuse from SiO_2_ mask toward NWs with decreased interval (larger diameter) [30,31]. Effective interval between the NW arrays (pitch) is regarded as dominant factor for the In incorporation rate. Therefore, the In content can likewise be increased by simultaneously decreasing the pitch and diameter. By optimizing the geometry and growth conditions, a broad emission range as larger as 150–200 nm is expected in a monolithic growth, which can involve the emission peak wavelength from approximate 420 nm to 620 nm. Although multi-color emission by NW structures is a promising prospect, monolithic integration growth by metalorganic chemical vapor deposition (MOCVD) presents a major challenge. In particular, the growth temperature sensitively affects both the crystalline quality and emission efficiency of formed NWs [32]. Inspired by the modified growth method of planar *a*-plane multiple quantum wells [33], it is expected that by raising and lowering the growth temperature of the barriers and wells, respectively, we could enhance both the In incorporation and the crystalline quality of coaxial GaInN/GaN NWs.

In this work, we comprehensively investigated multiple NW structures monolithically grown by MOCVD. Three aperture patterns were integrated into a single GaN/sapphire template by electron beam lithography. For multiple color emissions, coaxially aligned GaInN/GaN multiple quantum shell (MQS) NWs were selectively grown on the integrated-pattern template. In the modified growth method, the quantum-barrier was grown at high temperature (800 °C) to increase the crystalline quality of the coaxial GaInN/GaN structures, and the quantum wells were grown at low temperature to enhance the In incorporation without the risk of reducing the MQS quality. After characterization the morphology and optical features of the NW samples, we analyzed the effect of triethylgallium (TEG) flow rate, barrier growth temperature, and well growth temperature on the In incorporation in the MQS NWs.

## 2. Experimental Growth

**Figure 1:**
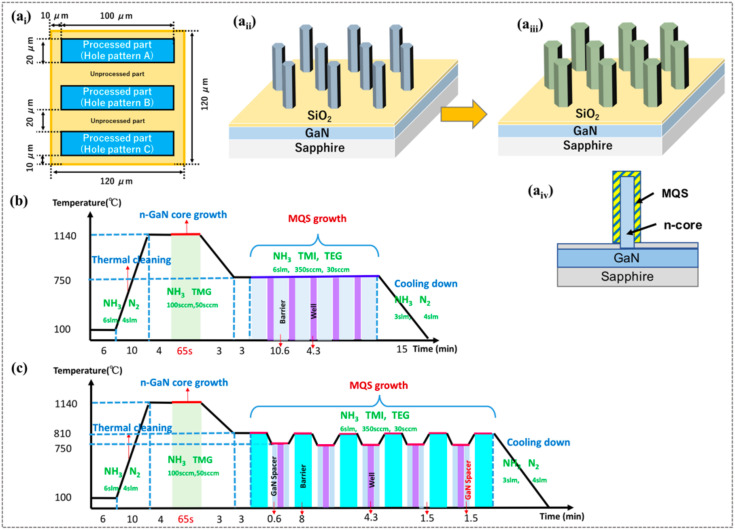
Schematics of (**a_i_**) the designed template with three aperture array patterns; (**a_ii_**) the n-GaN core; (**a_iii_**) the coaxially aligned GaInN/GaN multiple quantum shell (MQS) nanowires (NWs); and (**a_iv_**) cross section of a GaInN/GaN MQS NW. Schematics of the NW growth conditions at different barrier growth temperatures: (**b**) 750 °C and (**c**) 800 °C. The durations of each step are labelled in the horizontal axis.

The NW structures were selectively grown on patterned (0001) GaN/sapphire templates (Nanowin Co., Suzhou, China) by MOCVD. First, the aperture array patterns were formed using electron-beam (EB) lithography (JBX-6300FS, JEOL Ltd., Tokyo, Japan). The substrates were coated with a 30-nm thick SiO_2_ layer deposited by a magnetron sputtering system (CFS-4EP, Shibaura Mechatronics Co., Yokohama, Japan). Each (120 × 120) µm^2^ block was divided into three strip regions, while triangularly arranged aperture patterns with different pitches and hole diameters (1200 nm/400 nm, 880 nm/230 nm, and 800 nm/150 nm) were applied to the three strips, respectively, as specified in Figure 1(a_i_). After receiving an EB dose of 200 µC/cm^2^, the aperture patterns were formed by dipping into ZED-N95 development solution. The exposed patterns were transferred from the positive resist (ZEP520A) to the SiO_2_ mask layer by inductively coupled plasma (ICP) (CE-300I, ULVAC Co., Kanagawa, Japan) etching. The formed structures were cleaned in a 1:1 (30 mL/30 mL) mixed solution of H_2_SO_4_ and H_2_O_2_ to remove the residual polymer resist from the surface. The aperture structures were checked by a scanning electron microscope (SU70, Hitachi High-Technologies Co., Tokyo, Japan).

Panels (a_ii_), (a_iii_) and (a_iv_) of Figure 1 schematically show the grown n-GaN core NWs, the coaxial GaInN/GaN MQS structures on the n-cores, and the cross-sectional view of one GaInN/GaN MQS NW, respectively. Schematic diagram of the growth sequence in Figure 1b depicts the detailed growth conditions. The patterned template was pre-annealed in ambient ammonia (NH_3_, 6.0 slm) at 1140 °C under high pressure (90 kPa). Subsequently, the n-type GaN core NWs were monolithically grown on the three aperture patterns in a horizontal MOCVD system (SR 2000, Taiyo Nippon Sanso Co., Tokyo, Japan), operated in continuous flow mode. The height of the NWs was controlled via varying the growth time (55–65 s). Referring to the typical growth conditions on single-patterned templates [34], the growth temperature of the n-core was maintained at 1140 °C. During the n-core growth, the Ga and N were sourced from trimethylgallium (TMG) and NH_3_ precursors, respectively, at a fixed V/III ratio of 20. The silane (SiH_4_) doping precursor was injected at a rate of 2.79 × 10^−3^ μmol/min) with H_2_ carrier gas. The growth pressure was maintained at 90 kPa. Finally, five pairs of MQSs were sequentially grown at 750 °C, using trimethylindium (TMI) (350 sccm) and TEG (30–60 sccm) as the precursors of quantum well and quantum barrier growth, respectively (Figure 1b). During the MQS growth, the NH_3_ flow rate was kept at 5.0 slm. Figure 1c illustrates the growth sequence at a higher growth temperature (800 °C) for quantum barriers. To suppress the decomposition of the GaInN quantum wells, a thin GaN spacer shell (~2 nm) was inserted before and after the GaInN well growth at 750 °C. All the other growth conditions were as same as those with lower-temperature growth. Therefore, the grown GaInN quantum wells in are supposed to be approximate to that grown with low barrier temperature.

To investigate the effect of TEG flow rate, barrier growth temperature, and well growth temperature on the In incorporation of the MQSs, we prepared six NW samples (labeled as a–f in Table 1) with three different (pitches and diameters) combinations: p_1_ (1200 nm/400 nm), p_2_ (880 nm/230 nm), and p_3_ (800 nm/150 nm). To ensure similar heights of the n-core NWs, the growth times, and temperatures of the cores were slightly adjusted for each sample. Samples a and b were fabricated under different TEG flow rates (30 and 60 sccm) to investigate the effect of flow rate on the emission wavelength. Samples b and c were fabricated under the TEG flow rate of 60 sccm, but the barrier growth temperature was increased from 750 °C to 800 °C. Finally, to increase the In incorporation rate, the well growth temperature was varied from 750 °C to 710 °C for samples d, e, and f, respectively. The surface morphologies of the NW samples were inspected by SEM measurements, and the relative measurement error is less than ±5%. The optical properties of the coaxial GaInN/GaN NWs were characterized by CL measurements using a filter/detector (Gatan Mono CL4) equipped in the SEM system (SEM-SU5000, Hitachi High-Technologies Co., Tokyo, Japan). The probe current and accelerating voltage were set to 30 μA and 3 kV, respectively. The MQS structures grown by standard and modified sequence were inspected using a high-angle annular dark-field (HAADF) detector in Hitachi HD2700 STEM system (Hitachi High Technologies Co., Tokyo, Japan).

## 3. Results and Discussion

### 3.1. Morphology of the Monolithic Grown n-Core NWs

**Figure 2:**
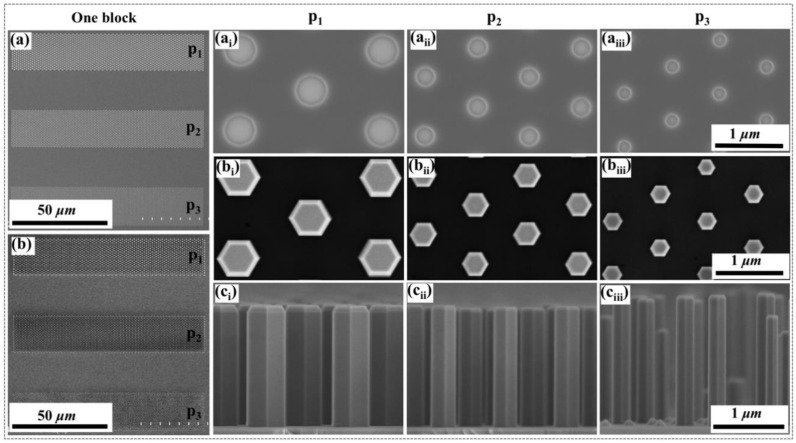
(**a**) SEM image of the template sample after EB lithography; Panels (**a_i_**–**a_iii_**) show the planar-view SEM images of the three aperture array patterns (p_1_, p_2_ and p_3_); (**b**) Planar-view SEM images of the sample after n-core NWs growth; (**b_i_**–**b_iii_**) Planar and (**c_i_**–**c_iii_**) cross-sectional view SEM images of n-core NWs in the patterns p_1_, p_2_ and p_3_, respectively.

After EB lithography and ICP etching, the dimensions of the aperture array patterns in the template samples were inspected by SEM characterization, as shown in Figure 2(a–a_iii_). As shown in Figure 2a, the strips were precisely aligned as desired, with 20-µm intervals. The diameters and pitches of the aperture holes in patterns p_1_, p_2_, and p_3_ are 511 nm/1.23 µm, 329 nm/898 nm, and 236 nm/809 nm, respectively. The diameters of the circular holes were much larger than that of designed, because they were enlarged by O_2_ ashing and the high lateral etching rate of ICP etching. Nevertheless, for all patterns, the GaN base inside the apertures was exposed to n-core NW growth, as shown in Figure 2b. No obvious parasitic growth or polycrystal deposition appeared on the SiO_2_ masks region. As confirmed in Figure 2(b_i_–b_iii_), uniform NWs are clearly observed in patterns p_1_, p_2_, and p_3_ with diameters of 535 nm, 360 nm, and 250 nm, respectively. Compared with the initial apertures, it reveals the low lateral growth rate of the NWs. The cross-sectional SEM images of the NWs in Figure 2(c_i_–c_iii_) show that n-cores grew perpendicularly to the substrate and developed a hexagonal prismatic shape. The growth rate was estimated to be 20 nm/s. Figure 2(c_iii_) shows a nonuniform height of NWs in pattern p_3_, because the desorption rate of Ga and N adatoms on smaller NW patterns (smaller filling factor) is more obvious due to the high growth temperature. The growth of uniform core NWs is quite sensitive to the growth temperature and geometry, while the optimal temperature slightly decreases with a decrease in diameter owing to the high desorption rate [35]. However, it is possible to monolithically uniform grow NWs in the entire patterns by controlling the growth temperature of the n-core NWs.

### 3.2. Effect of TEG Flow Rate on Emission Wavelength

**Figure 3:**
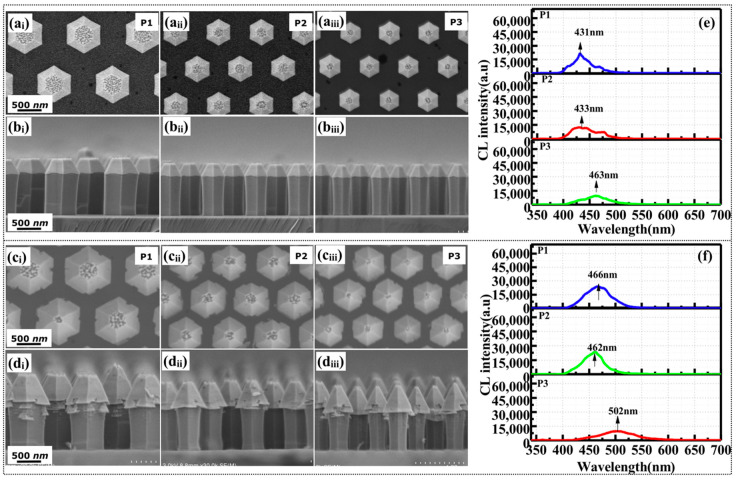
(**a_i_**–**a_iii_**) Planar and (**b_i_**–**b_iii_**) cross-sectional SEM images of sample a grown under a low TEG flow rate of 30 sccm; (**c_i_**–**c_iii_**) Planar and (**d_i_**–**d_iii_**) cross-sectional SEM images of sample b grown with a high TEG flow rate (60 sccm). CL spectra measured at the bottom areas of NWs in patterns p_1_, p_2_, and p_3_ of (**e**) sample a and (**f**) sample b.

GaInN/GaN MQS structures were sequentially grown on the n-core NW structures. Two NW samples a and b were prepared at different TEG flow rates of 30 sccm and 60 sccm, respectively. Planar and cross-sectional SEM images of the three patterns of both samples are shown in Figure 3. Two dominant facets (semi-polar and non-polar planes) are observed in all patterns. The heights of the non-polar region in NWs are uniform over the three patterns, while that of apex area (semi-polar plane) gradually decreases with a decrease in diameter. More adatoms were impinged and contributed to the growth on the *c*-plane area in pattern p_1_ with larger diameter, resulting in the higher growth rate along *c*-direction. In sample a, the diameters of the NWs (bottom part) in patterns p_1_, p_2_, and p_3_ are 650 nm, 446 nm, and 372 nm, respectively. In sample b, grown under a higher TEG flow rate than sample a, is expected to show an increased MQS growth rate. However, the diameter was only enlarged at the top part of the NWs, and many flakes were formed at the junction between the semi-polar and non-polar planes, as shown in Figure 3(d_i_–d_iii_). Typically, the impinged precursors on the top area of the NWs mainly contribute to the growth at the top area [34,36]. The flakes may have been formed by the high growth rate at the apex and the overflow of precursors under the higher TEG flow rate, which can reduce the diffusion of impinged adatoms toward the bottom area. As a result, the thickness of MQS in the bottom area is just slightly larger than that of sample a. Simultaneously, more Ga adatoms diffused toward the sidewalls of the NWs from SiO_2_ mask, which may promote the In incorporation on non-polar planes. It was concluded that at the high TEG flow rate, excessive precursors were supplied to the top area of the NWs, causing abnormal growth. In addition, as the In adatoms have a shorter diffusion length than that of Ga adatoms [37], the flakes formed between the semi-polar and non-polar planes are generally In-enriched.

The CL spectra acquired from the bottom areas of the NWs in patterns p_1_, p_2_, and p_3_ of samples a and b are depicted in Figure 3e,f, respectively. The emission peaks exhibit a redshift as the diameter (pitch) decreased from patterns p_1_ (431 nm) and p_2_ (433 nm) to p_3_ (463 nm). This trend was ascribed to the higher incorporation probability in the patterns with smaller diameter and pitch, as a result of the enhanced contribution from lateral diffusion (in pattern p_3_). Under higher TEG flow rate, the emission peaks of the NWs on all patterns were further redshifted by ~30 nm. This confirms that the higher TEG flow rate promoted the diffusion and suppressed the desorption rate of In adatoms, because the exposed surface to the atmosphere was immediately covered by Ga adatoms [38]. This phenomenon is more obvious in the NWs with small diameter and pitch (<800 nm) in pattern p_3_, wherein the dimension is smaller than the diffusion length (<800 nm). Compared with the spectra of sample a, the CL intensity of the NWs was slightly stronger in sample b, owing to the slightly thicker quantum wells formed with the higher TEG flow rate. Therefore, increasing the TEG flow rate effectively induces a redshift of the peak wavelength. However, the sample b fabricated under the larger TEG flow rate also contains In-rich flakes at the top area, leading to a degradation of the crystalline quality at the top part of the NWs.

### 3.3. Improving Crystalline Quality by Raising the Growth Temperature of Quantum Barriers

**Figure 4:**
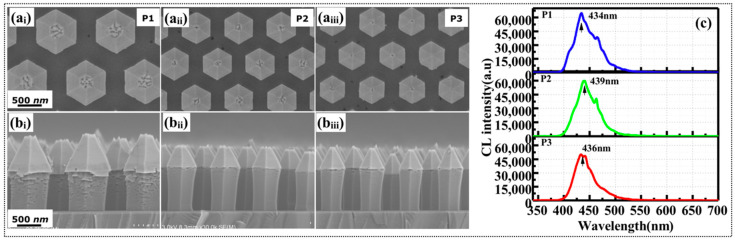
(**a_i_**–**a_iii_**) Planar and (**b_i_**–**b_iii_**) cross-sectional SEM images of the NW sample c formed at a barrier growth temperature of 800 °C; (**c**) CL spectra measured at the bottom areas of the NWs on patterns p_1_, p_2_, and p_3_.

As mentioned above, increasing the TEG flow rate increased the In incorporation rate, despite causing abnormal growth in the top areas of the NWs. High temperature is known to promote the In decomposition rate. To suppress the formation of In-rich flakes, the growth temperature of the quantum barriers was increased from 750 °C to 800 °C for sample c, while the other growth conditions were those of sample b. The effects of higher barrier growth temperature on the morphology and CL emission properties were then investigated. Figure 4 shows the SEM morphology and CL spectra of the NWs on patterns p_1_, p_2_, and p_3_. On all patterns, the morphologies of the NWs were clearly improved in comparison to that of sample b. The formation of In-rich flakes was suppressed by the high decomposition rate at high growth temperature, but a few flakes still appeared on the NWs of pattern p_1_. Figure 4c presents the CL spectra measured at the bottom parts of the NWs on patterns p_1_, p_2_, and p_3_, where the scale is same as that of Figure 3. The CL intensities of the NWs on each pattern were 2–3 times stronger than that of sample b. Such enhancement indicates that increasing the barrier growth temperature by 50 °C certainly improved the crystalline quality of the GaInN/GaN MQS structures. Nevertheless, the emission peaks of the NWs on patterns p_1_, p_2_, and p_3_ were blue-shifted to 434, 439, and 436 nm, respectively. Ramping up of growth temperature inevitably increased the decomposition of the In-rich flakes and the desorption of In from the GaInN quantum wells.

### 3.4. Enhancing Emission Wavelength by Lowering the Growth Temperature of Quantum Wells

**Figure 5:**
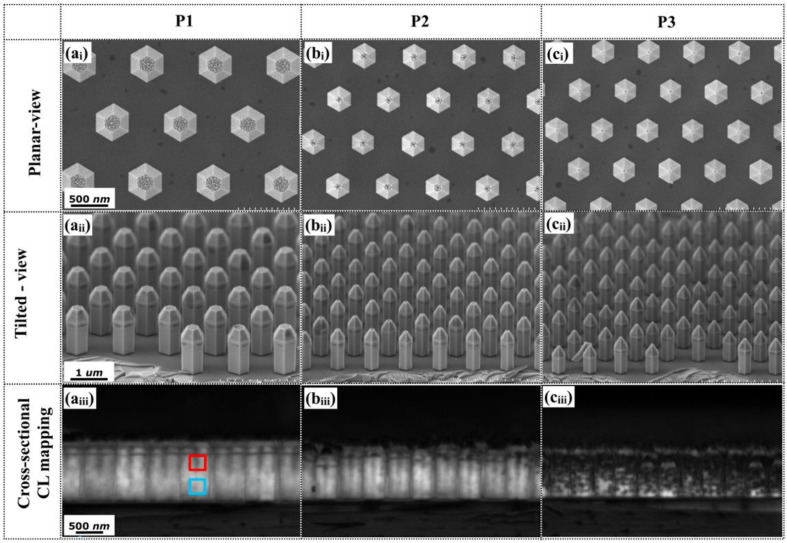
(**a_i_**–**c_i_**) Planar and (**a_ii_**–**c_ii_**) tilted-view SEM images of patterns p_1_, p_2_, and p_3_ of NW sample e, grown at a well temperature of 730 ℃; Panels (**a_iii_**–**c_iii_**) are the corresponding panchromatic CL mappings.

In sample c grown at barrier temperature of 800 °C, the crystalline quality of MQS structures was much higher than in sample b with a barrier growth temperature of 750 °C, despite of the similar emission wavelength in three different patterns. To broaden the emission-wavelength range in the monolithically grown NW samples, it is important to carry out a further investigation of the quantum well growth temperature. Samples d, e, and f were fabricated with well growth temperature of 750 °C, 730 °C, and 710 °C, respectively, and the effect of the well growth temperature on the emission wavelength was investigated. Figure 5(a_i_–c_i_,a_ii_–c_ii_) shows the planar and tilted-view of SEM images of patterns p_1_, p_2_, and p_3_ of NW sample e, fabricated at a well growth temperature of 730 °C. With a high barrier growth temperature, uniform NWs were simultaneously achieved on patterns p_1_, p_2_, and p_3_ of sample e. With standard growth sequence, the formation of In-flakes usually occurs as the MQS growth temperature decreases [34]. Here, In-flakes are absent in the SEM characterizations of sample e, which was also confirmed in the other two samples grown at 750 °C and 710 °C. This consolidates that increasing the growth temperature of the GaN barriers can compensate the degradation of the MQS crystalline quality (morphology) at low growth temperatures of the GaInN wells [34]. Such an improvement is attributable to the unintentionally induced thermal annealing process of the GaInN wells during barrier growth. The corresponding panchromatic CL mappings of the cross-sectional configuration are depicted in Figure 5(a_iii_–c_iii_). The CL emission was spatially uniform across the NWs on patterns p_1_ and p_2_. However, the NWs on pattern p_3_ (with a smaller pitch than the other patterns) contained many weak emission points, possibly where In-enriched clusters had formed. The average diameters of the NWs in patterns p_1_, p_2_, and p_3_ are 588 nm, 406 nm, and 390 nm, so the estimated thicknesses of GaInN/GaN MQS are around 26.5 nm, 23 nm, 70 nm, respectively. It is clear that the growth rate on pattern p_3_ is much higher, resulting in an enhanced In incorporation rate and degradation of In homogeneity.

**Figure 6:**
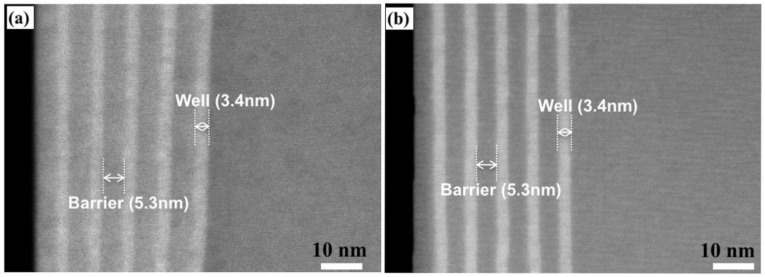
STEM-HAADF image of InGaN/GaN structures grown on single pattern (pitch—1200 nm, diameter—300 nm) by using (**a**) standard and (**b**) high temperature of quantum barriers, respectively.

To confirm the growth of GaInN/GaN MQS structures, the NWs grown on single pattern with similar pitch (1200 nm) and diameter (300 nm) to that of pattern p_1_ were characterized by STEM measurements. Figure 6 shows the STEM-HAADF images of the MQS structures grown with standard and high temperature of quantum barriers, while the growth conditions are totally same as that for samples a and d, respectively. The thickness of GaInN quantum wells are virtually identical in these two samples, which confirms that increasing the barrier temperature to 800 °C did not cause additional damage to the wells. The interface between GaInN well and GaN barriers are more distinguishable in the sample grown at higher barrier temperature. Thus, compared with sample a, the crystalline quality of MQS structures was improved in sample d by increasing barrier growth temperature.

**Figure 7:**
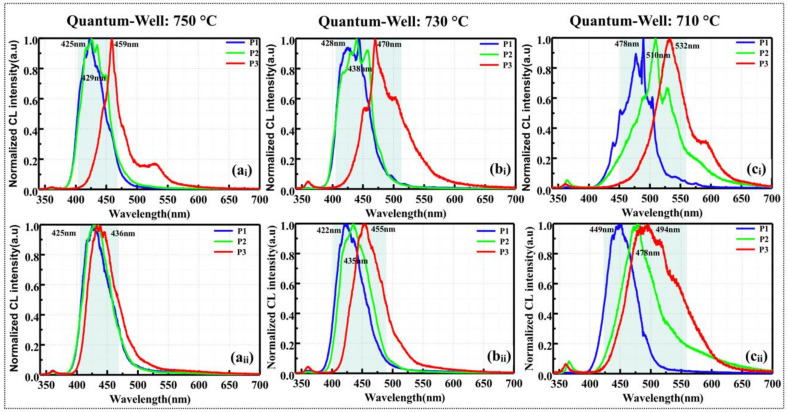
Normalized CL emission spectra of the top areas of the NWs in samples d, e, and f grown with different well temperatures of (**a_i_**) 750 °C; (**b_i_**) 730 °C; and (**c_i_**) 710 °C, respectively. The bottom panels are the normalized CL spectra measured at the bottom areas of the corresponding NWs.

CL measurements were performed at the top and bottom areas of the NWs on samples d, e, and f, respectively. The peak intensities of patterns p_1_ and p_2_ in these samples are stronger than that in reference sample a (p_1_—23,000 a.u., p_2_—13,000 a.u) owing to the improved MQS quality. Compared to patterns p_1_ and p_2_, a similar weaker emission was also observed in pattern p_3_, especially in sample f. Figure 7 shows the normalized CL spectra acquired at the top and bottom areas of NWs on all patterns. Since the flow rates of precursors and growth time were identical in the same patterns of samples d, e, and f, a similar thickness of GaInN quantum well was expected in NWs with same diameters. Therefore, the quantum confinement induced peak-shift by the variation of GaInN thickness can be excluded in the patterns with same diameters. Even though it is not able to accurately estimate the In fraction from CL emission peaks, the variation trend of the In incorporation can be identified in the patterns. Here, it further testifies the redshift of the NW emissions from patterns p_1_ to p_3_, which is due to the increased In incorporation probability as the filling factor (diameter/pitch) decreases. Notably, the broader emission in pattern p_3_ (with thicker MQSs) is due to the high In content and slight degradation of In homogeneity. When the pitch of the patterns is small, the distance between the NWs is also relatively short (approximating the diffusion length of the In adatoms), and the number of precursors incorporated by diffusion from the SiO_2_ mask layer overcomes the desorption loss.

The spectra measured at the top and bottom areas reveal that the peak wavelengths are red-shifted with a decrease in the growth temperature of the GaInN wells, as shown in Figure 7(a_i_–c_i_,a_ii_–c_ii_), respectively. When grown at lower well temperatures, the multi-color emissions from patterns p_1_, p_2_, and p_3_ of the NWs covered a broader range with a larger full-width-at-half-maximum (FWHM) than that at the higher well temperature. Basically, the In incorporation gradually increases in the outer shells owning to the increased diameter (decreased interval between NWs), as confirmed in our previous work [39]. As mentioned in Section 3.3, the high growth temperature of barriers accelerates the In decomposition, but a low well growth temperature is expected to increase the In incorporation rate. In addition, the CL spectra of samples d, e, and f show a slight blueshift from the top to bottom areas of NWs. As the NWs are periodically adjacent to each other, there were lower density of precursors supplied to the SiO_2_ mask layer between the adjacent NWs and the bottom area of the NWs. Therefore, the amount of incorporated In in the top areas of NWs was higher than at the bottoms, thereby broadening the emission wavelength. Nevertheless, the diffusion length of adatoms also increased with an increase in well temperature, so more adatoms can diffuse toward the bottom of NWs, leading to a uniform spatial distribution of emission wavelength. As shown in Figure 7, the well growth temperature of the MQSs, the height of the NWs, and the filling factor (pitch) of the patterns all influenced the peak wavelength of the NWs. Among these results, the longest emission wavelength was 532 nm from the NWs of pattern p_3_ grown at a well growth temperature of 710 °C.

The emission wavelength is expected to extend beyond 532 nm by further optimizing the pattern geometry. Since the optimal temperature for NWs with different diameter is different, the feasibility of monolithic growth depends on the combination of diameters and effective pitch. Therefore, the diameter of the aperture hole around 600–150 nm is reliable for both nanoimprint and monolithic growth with approximate heights. In addition, the pitches of the combined patterns can be optimized for longer or shorter wavelength emission, because the In incorporation rate also related to the probability of diffused precursors from SiO_2_ mask toward NWs. Ultimately, white light emission can be achieved by combining the multi-color emissions from the monolithically grown coaxial GaInN/GaN MQS NW structures.

## 4. Conclusions

In this study, the selective-monolithic growth of coaxial GaInN/GaN NWs with three different diameters and pitches was investigated by changing the TEG flow rate, barrier and well growth temperatures during MQS growth. Increasing the TEG flow rate increased the probability of In incorporation, and redshifted the emission wavelength of each (pitch/diameter) aperture pattern (p_1_, p_2_, and p_3_) by approximately 30 nm. However, the crystalline quality was degraded by In-rich flakes formed on the upper parts of the NWs. The growth sequence was modified by increasing the growth temperature of the quantum barriers, thereby eliminating the abnormal growth at the top of the NWs. As a result, the improved crystalline quality boosted the CL emission intensity by 2–3 times, despite of the higher In decomposition rate. Furthermore, the occurrence of In desorption in this modified growth sequence was suppressed by decreasing the growth temperature of the quantum wells from 750 °C to 710 °C. Lowering the well growth temperature significantly broadened the emission wavelengths of the three NW patterns, especially those of the smallest pattern p_3_. In addition, the low growth temperature slowed growth and diffusion rate of In adatoms, yielding a higher In incorporation rate and thereafter a redshift of peak wavelength. The pitches of the integrated three patterns can be further optimized for a broader emission wavelength, because the In incorporation rate also related to the probability of diffused precursors from SiO_2_ mask toward NWs. Therefore, the multiple color emissions from monolithically grown coaxial GaInN/GaN NWs can potentially realize NW-based white/micro-LEDs.

## Figures and Tables

**Table 1:** Metalorganic chemical vapor deposition (MOCVD) Growth Parameters of the NW Samples.

Sample Name	Pattern	Pitch (nm)	Diameter (nm)	Height (µm)	TEG Flow Rate (sccm)	Barrier Growth Temperature (°C)	Well Growth Temperature (°C)
a	p_a1_	400	1200	1.4	30	750	750
	p_a2_	230	880	1.2			
	p_a3_	150	800	1.2			
b	p_b2_	400	1200	1.6	60	750	750
	p_b2_	230	880	1.5			
	p_b3_	150	800	1.3			
c	p_c1_	400	1200	1.6	60	800	750
	p_c2_	230	880	1.3			
	p_c3_	150	800	1.3			
d	p_d1_	400	1200	1.1	30	800	750
	p_d2_	230	880	0.9			
	p_d3_	150	800	0.9			
e	p_e1_	400	1200	1.4	30	800	730
	p_e2_	230	880	1.2			
	p_e3_	150	800	1.0			
f	P_f1_	400	1200	1.4	30	800	710
	P_f2_	230	880	1.1			
	P_f3_	150	800	1.0			
